# Venom and Social Behavior: The Potential of Using Spiders to Evaluate the Evolution of Sociality under High Risk

**DOI:** 10.3390/toxins13060388

**Published:** 2021-05-28

**Authors:** Laura Gatchoff, Laura R. Stein

**Affiliations:** Department of Biology, University of Oklahoma, Norman, OK 73019, USA; laura.stein@ou.edu

**Keywords:** arachnid, cannibalism, *Latrodectus*, learning, risk assessment, social risk, venom

## Abstract

Risks of sociality, including competition and conspecific aggression, are particularly pronounced in venomous invertebrates such as arachnids. Spiders show a wide range of sociality, with differing levels of cannibalism and other types of social aggression. To have the greatest chance of surviving interactions with conspecifics, spiders must learn to assess and respond to risk. One of the major ways risk assessment is studied in spiders is via venom metering, in which spiders choose how much venom to use based on prey and predator characteristics. While venom metering in response to prey acquisition and predator defense is well-studied, less is known about its use in conspecific interactions. Here we argue that due to the wide range of both sociality and venom found in spiders, they are poised to be an excellent system for testing questions regarding whether and how venom use relates to the evolution of social behavior and, in return, whether social behavior influences venom use and evolution. We focus primarily on the widow spiders, *Latrodectus*, as a strong model for testing these hypotheses. Given that successful responses to risk are vital for maintaining sociality, comparative analysis of spider taxa in which venom metering and sociality vary can provide valuable insights into the evolution and maintenance of social behavior under risk.

## 1. Introduction

Many social species benefit from cooperation, though there are an assortment of risks associated with social behaviors, such as disease spread [1], predation threats [1], and conspecific competition [2,3]. The definition of sociality fluctuates, but Wilson’s definition of sociality as the tendency of conspecifics to live cooperatively in groups is currently widely accepted [4]. This paper will consider social behavior as any type of interaction between or among conspecifics. Conspecific competition over resources such as food and mates can result in anything from mild inconvenience to the death of the individual [5,6].

Many species deal with social risk through engaging in multiple social interactions and adjusting their responses accordingly. There are many different mechanisms involved in this behavioral modification. When an individual “loses” an agonistic social interaction, hormonal changes in the brain may cause changes in behavior that make the individual more likely to lose in a future interaction [4]. Likewise, the “winning” individual experiences physiological changes that may result in this individual being more likely to continue to win in future interactions, a phenomenon known as the “winner/loser effect” [7]. These mechanisms can help to establish social hierarchy and/or to create a cohesive social environment.

Sociality’s delicate balance of risk and reward is present in invertebrates as well as vertebrates, and some of the most highly studied cases of invertebrate sociality include the eusocial invertebrate systems such as order Hymenoptera (bees, wasps, ants, etc.). These eusocial organisms live in a highly specialized caste system, with only one colony member reproducing and others raising the offspring [8]. Eusocial individuals can recognize members of their colony [9], communicate with members of their colony [10], and can learn from observing other colony members [11,12,13]. Eusocial species also engage in collective defense of the colony against external threats. It has been hypothesized that the key to the evolution of eusociality is partially in this defense, through the evolution of venomous stingers [14]. This hypothesis has received some support via the scarcity of non-venomous eusocial hymenopterans and presence of non-venomous solitary hymenopterans [14], though termites are both non-venomous and eusocial. Another intriguing piece of evidence for the correlation between venom and social evolution in invertebrates is the evolution of immune-attacking secretions in eusocial aphids, which have many venom-like properties and could be a precursor to venom [15].

While Hymenopterans are widely lauded as model systems for the study of the evolution of sociality, one difficulty in using this group for comparative studies on the evolution of social behavior lies in the few independent origins of sociality, with only seven to eight instances across all social hymenopterans [16]. Spiders, like social insects, show the nearly full range of social categories, and sociality has been estimated to have evolved independently 15–16 times [17]. Recent comparative genomic work suggests intriguing convergence of molecular evolution in the development of sociality [18]. As the majority of spider groups are venomous, this group provides a promising foundation for examining potential links between the evolution of venom and sociality in concert with Hymenopteran studies. Yet, while highly social spiders are well-studied, we know little about the properties of their venom, whether/how its molecular components and function differ from less social spiders, and whether spider venom components and/or functions change over evolutionary transitions.

Researchers have proposed an index of sociality across four broad categorical levels: solitary, subsocial, social, and eusocial [19]. This way of categorizing sociality qualitatively categorizes species based on various behaviors and aspects of natural history [19]. Although this categorization is based on a linear continuum or stair-step model, this is not necessarily the case in nature, as some of the different “levels” could actually occur across separate evolutionary directions. For example, subsociality could be an evolutionary end and not just a step on the way to eusociality [20]. While this categorization accurately describes many species, many others fall between these categories, or show indications of sociality that may not match specifically the descriptors that make up the category definitions [19]. These in-between species are often less researched than their counterparts, but may represent important transitions in the evolution of social systems.

One group of organisms that lies between categories is the widow spider (*Latrodectus* spp.). Members of the genus *Latrodectus* are considered facultatively social, live in large juvenile groups from hatching through two weeks following emergence from the egg sac, and adult females aggregate outside of the breeding season [21]. Group living in these systems is dependent on nutritional state, prey availability, and population density [21]. Communally living juveniles and females frequently cannibalize their conspecifics, including kin [21,22]. To survive in this high-risk social environment, *Latrodectus* individuals must recognize social risks and alter their behavior in response. While individuals in many taxa adjust their responses based on outcomes of social interactions [23], we understand little about the social ecology of venomous, cannibalistic species such as *Latrodectus,* where negative interactions could result in death. These extreme consequences, together with *Latrodectus*’s facultative sociality, make *Latrodectus* an excellent system to study responses to social risk in comparison with genera having more highly social systems (see Section 2 for examples). By exploring how individuals respond to social risk in a high-risk environment where consequences are amplified, we can reach a greater understanding of causes and consequences of social behavior. Additionally, members of this genus are highly venomous and lie between traditional stair-steps of social categories. This system, along with comparisons to other spiders, provides an excellent opportunity to test hypotheses relating the evolution of social behavior and venom. This review will focus primarily on non-eusocial arachnids and discuss the literature examining how cannibalistic, venomous taxa such as *Latrodectus* adapt to the inherent risks of social interaction, and how evolution of venom may be intricately intertwined with social behaviors.

## 2. Sociality in Arachnids

Arachnids are typically known for being solitary, but this assessment has key exceptions [24]. Juveniles of certain amblypygid species have been observed aggregating and associating with their mothers [24], and some burrowing scorpions (*Heteromeptrus fulvipes*) have been witnessed dividing labor and sharing food [25]. Burrowing scorpion sociality possibly evolved due to the necessity of burrows for predator avoidance and protection from dehydration. Juvenile scorpions struggle with burrow construction, and sharing may increase survival [26], suggesting an important role for juvenile social environment. In contrast to expected cannibalism among juveniles in the scorpions (see Section 4.1.2 for more information on sibling cannibalism), no cannibalization was observed in the lab [25].

### 2.1. Differences between Solitary and Social Spiders

Sociality in spiders is divided into two main classifications [27]: “cooperative” species, also referred to as “quasi-social” [28], which share duties such as care of young and foraging, and “communal” species, which reside in aggregations but undertake all duties alone and do not engage in maternal care [29]. More recent terminology classifies cooperative species as “non-territorial permanent-social”, and communal species as “communal-territorial” or “territorial permanent-social” [29]. While most spiders are considered solitary, there are at least 53 known species of communal-territorial spiders [30] and 25 of non-territorial permanent-social [17]. No social spiders have been identified as eusocial [31]. Sociality in spiders has numerous independent evolutionary origins [17], likely associated with collective defense and hunting abilities [32], but any concurrent changes in venom properties or use along with these evolutionary transitions remain understudied. Recent genomic comparisons among social, subsocial, and solitary spiders found mainly differences in genes associated with immunity and metabolism, supporting hypotheses relating social evolution in spiders to hunting and defense, but genomic and behavioral studies and data in spiders across varying social structures remain scarce [18].

Although only certain species are labeled as “social spiders,” spiders, like other arachnids and other taxa, follow a spectrum of sociality not limited to just social spiders and solitary spiders. *Latrodectus*, for example, is not classified as a social spider genus by the aforementioned papers, but exhibits several known social behaviors. *Latrodectus* adult females are known to aggregate with juveniles and other females during the fall and winter, dispersing only in the spring and summer in time for mating season [21,33] Additionally, *Latrodectus* juveniles remain in groups for at least two weeks prior to dispersal and have been observed sharing food [34].

Both social and solitary spider taxa show evidence of kin recognition. Juvenile cannibalistic social spiders preferentially attack non-related juveniles over related juveniles in the lab, only cannibalizing the latter when the alternative is starvation [35,36,37]. Even fully solitary wolf spiders [38] and amblypygids [39] are less likely to prey on kin than on other unrelated individuals. Evidence for kin recognition is seen in facultatively group living *Latrodectus* as well. Juvenile *Latrodectus* are more likely to cannibalize nonrelated individuals in studies conducted in the lab, and sometimes will completely decline to cannibalize kin [40,41]. Pheromones associated with silk have been found to play an important role in providing kin recognition cues in both social and solitary spiders [42,43,44,45,46,47]. Males use silk pheromones to find females in many *Latrodectus* species [48,49], and therefore chemical cues seem like a promising mechanism for kinship recognition in this group, although this has yet to be formally tested. Alternatively, *Latrodectus* may not use chemical cues: developmental synchrony prevents cannibalism in *L. hesperus*, suggesting a pathway for familiarity over chemical kin recognition in forming cannibalism decisions [50]. Untangling these non-mutually exclusive mechanisms can help shed light on cannibalistic decision-making and, ultimately, group dynamics in subsocial species.

Given the prevalence of kin recognition among cannibalistic social arachnids and some non-social arachnids, kin recognition likely contributes to the evolution of sociality in cannibalistic species. Interestingly, many colonies of social spiders are highly inbred, despite the evolutionary cost of inbreeding [51,52]. Whether this is because of proximity to other colonies or a consequence of kin selection remains under discussion. However, interaction of any type between colonies is rare, increasing support for the role of kin selection in cannibalistic sociality [52]. Further research into the transition from outbreeding to inbreeding in social spiders could provide valuable insight into the mechanisms behind the evolution of sociality in cannibalistic and/or venomous species.

One potential differentiation between solitary and social spiders lies in venom properties. In Hymenoptera, the venom of solitary and social wasps differs both in content and use [53,54]. Solitary wasps mainly use venomous stings for prey capture, stinging to paralyze. In social wasps, stings are used primarily for defense of the colony, providing evidence for the theory of venom evolution as a function of defense to facilitate the evolution of sociality [54]. Unfortunately, there are no comparative analyses of venom contents in social and solitary spiders. However, one intriguing example exists of a communal spider taxon that is entirely non-venomous [55], suggesting that venom may potentially have been lost in conjunction with the evolution of communal living. Whether venom in spiders is a pathway for evolution of sociality, or the lack of venom is an adaptation to sociality, the possibility of a connection between venom and social evolution remains an unknown but intriguing area of study.

## 3. Risk Assessment

Individuals must assess risk from predators, prey, and conspecifics, and then make decisions based on that assessment. Web-spinning and hunting spiders both learn to identify and react to potentially dangerous venomous prey, such as ants or wasps [56]. Spitting spiders, for example, use venom both in hunting and for defense. They are risky to attack from the front due to their spitting behavior, in which they rapidly eject a mixture of venom, silk, and glue at the predator, prey [57], or even occasionally kin [58]. Additionally, spitting spiders regulate their spit expenditure in response to more difficult prey or more risky situations, similar to how many other spiders regulate their venom expulsion (Section 3.1) [58]. However, contrary to the similarity of spitting expenditure to venom expenditure, and despite the spit containing venom components, the spit does not appear to have any toxic effects on invertebrates, despite previous thought [58]. Some peptide components of the venom have been described, but no receptors have been identified as of yet, and the function of the known peptides remains unclear [59].

Spiders are known to behaviorally modify attacks based on risk assessment of prey. *Portia* jumping spiders, for example, will only attack spitting spiders from the back, with one key exception. If the spitting spider is carrying an egg sac, *Portia* spiders will attack it from the front, since it cannot spit [60], suggesting that *Portia* avoid the spitting defense despite venom from the spit not having toxic effects on invertebrates [44]. Web spiders similarly adapt to risky prey, learning through experience to identify which vibrations on their web signify a high-risk prey item, such as an ant, and avoiding those vibrations [61]. Although web-spinning spiders will occasionally catch risky (typically venomous) prey, in a field count only about 10% of prey caught by web-spinning spiders was classified as “risky”, e.g., orders Araneae (spiders) and Hymenoptera (wasps) [62]. This low percentage is potentially due to learned avoidance behavior. While many risks that spiders face come from either predators or their prey, in social situations spiders face cannibalism from their conspecifics, placing them in the interesting position of being at once predator preying on risky prey and potential risky prey themselves. For sociality to be feasible in these cannibalistic taxa, spiders must be able to assess and respond to social risk. As risk assessment in both predator and defense contexts has been demonstrated across multiple spider taxa [50,51,52], it is possible that spiders engage in similar risk assessment in social interactions, providing the foundations needed to evaluate whether and how venom influences social behavior and vice versa.

### 3.1. Venom Metering

When faced with risks, spiders have a series of choices, typically to play dead, run, or attack [42]. Of these, attacking is potentially the costliest. A spider can attack with webbing or venom. Both of these are presumably costly to produce, and venom in particular is very complex in composition [63,64]. In part because of these costs, venomous organisms typically meter their venom use, injecting only a minimal or optimal amount for each defensive or offensive encounter [65,66]. For example, in lab-based studies, rattlesnakes of all ages injected more venom into larger prey than into smaller prey, after being exposed to prey of various sizes [67,68]. Spiders are no exception, also capable of venom metering. Juvenile *Latrodectus hesperus* spiders relied primarily on evasive behaviors in response to predator cues in the lab, switching to more aggressive behaviors such as biting and presumed venom expulsion as they grew older and bigger, though it is not certain whether this difference was caused by age or size [69]. In the aforementioned *L. hesperus* study, spiders tested repeatedly as adults acclimatized to the predator cues, with more experienced spiders preferring more evasive behaviors than aggressive behaviors [69]. This indicates that both age/size and experience play a role in *L. hesperus* venom metering, with larger and less experienced spiders relying more heavily on combative behaviors than smaller or more experienced spiders. When *Latrodectus* spiders do bite, they often administer “dry bites” (bites without venom) when defending themselves against humans or other potential threats, and the amount of venom they inject is directly correlated with the threat level [70]. Some spiders such as hunting spiders (*Cupiennius salei*) adjust their venom based on olfactory cues and the behavior or perceived threat level of their prey, not simply the prey size [63]. Additionally, ontogenetic shifts in venom profiles and toxin abundance from juvenile to adulthood and after have been observed in tarantulas, directly relating to size [71], and some differences in venom composition have been shown to exist in juvenile and adult *Latrodectus* [69], although these differences have yet to be formally described. Despite these examples, few ontogenetic studies have been conducted in *Latrodectus* or closely related spiders [71,72], representing a fruitful area of study for understanding the evolution and function of spider venom. Changes in venom composition during ontogeny may relate to behavioral changes, including venom metering. Taken together, these findings suggest that age, experience, and cue type may all influence venom metering in spiders across multiple contexts, such as prey capture, predator defense, and perhaps social interactions such as cannibalism.

## 4. Social Risk

### 4.1. Cannibalism

Cannibalism is often associated with spiders, especially *Latrodectus*, and is one of the major risks that individual spiders face in social interactions. Cannibalism can occur between individuals of either sex and any age [73,74]. Adults and juveniles of some invertebrates gain nutrients by eating unhatched eggs, though adult females sometimes show preference for eggs laid by other females, possibly indicating kin recognition [75]. Conversely, mother spiders sometimes offer themselves up as a meal for their offspring, providing nutrients for the next generation in a process called “matriphagy” [76,77]. Old or weak spiders will also often end up as a meal for their younger, healthier relatives [76,78].

Current research on arachnid venom metering focuses on responses to predator or prey stimuli, however, little is known about venom metering in cannibalistic interactions [66]. Cannibalism could easily be interpreted as interactions with prey or interactions with a predator, so it is difficult to draw inferences from current research. However, certain defensive or aggressive tactics such as increased venom or bites based on aggression level of the other spider likely still apply. For example, many cannibalizations occur with a larger spider attacking a smaller spider [50]. In this situation, the smaller spider may display defensive rather than aggressive strategies, resulting in less risk for the cannibalizing spider. The cannibalizing spider’s reaction to more aggressive strategies from the smaller spider is unknown, as there is little research on the details of how such cannibalizations outside of mating contexts occur. Understanding behaviors occurring during cannibalism can help shed light on the use and evolution of venom in response to conspecific behaviors, and whether and how they differ from standard foraging or defensive behaviors.

Research on cannibalism in spiders focuses nearly entirely on the cannibalistic behavior, and seldom addresses the use of venom, assuming that females (and other cannibalizing spiders) use venom during cannibalism as they would against prey. Conversely, in one species of spider, the male bites the female during courtship, paralyzing her to prevent any resistance [79]. The effect of this male spider’s venom on the female indicates that spiders are susceptible to venom of their own species. However, venom is known to differ between male and female scorpions, and female paralysis in courtship might be simply a sexual adaptation [80]. More research is needed to determine the use of venom during cannibalism, and the effectiveness of venom on spider conspecifics, to allow testing of hypotheses regarding venom and evolution of social behavior.

#### 4.1.1. Courtship and Mating

Risk assessment research in *Latrodectus* typically focuses on male mate choice, since sexual cannibalism occurs frequently. All species in *Latrodectus* engage in sexual cannibalism, but males invite cannibalism in only three documented species, *L. mirabilis* (Argentinian black widow) (L. Barrufaldi, *personal communication), L. hasselti* (redback spiders) and *L. geometricus* (brown widow spiders) [81]. Males of the latter two species engage in a “somersault” behavior in which the males present their abdomen to the females, leading to the males’ death and consumption [82,83]. Although this might seem to be the opposite of risk mitigation, self-sacrifice increases the time the female spends copulating [84], which is directly related to paternity [85]. Despite the male’s willingness, however, the female will not always consume the male, likely due to her hunger state [86]. It can be difficult for males to find mates, and so it is less risky for a male to give the ultimate investment to one female than to search for a second mating opportunity [87]. Since females disperse from their colonies and live solitarily during mating season [33], males may or may not be able to find other females. The male can either take the risk of cannibalism with the benefit of a guaranteed mate, and invest himself fully in the offspring from that mating, or he can try to find another mate, with the risk of dying before he can do so. In addition, the journey itself to find another female can be particularly dangerous. Field studies on both *L. pallidus* and *L. hasselti* indicate high mortality rates for journeying males [87,88].

While males of self-sacrificial species do not appear to take action to reduce the risk of sexual cannibalism, many males in other species, such as *L. hesperus*, that are able to mate multiple times, will [89]. The first male to mate with a female is likely to get sperm precedence over males who mate with the female later [90]. To be the first male to mate with a female, males will sometimes mate with subadult females that are about to molt to adulthood, puncturing the exoskeleton above the newly formed genitalia. Subadult females are less likely than adult females to engage in sexual cannibalism, for reasons that are currently unknown. Despite this reduced cannibalism risk, males do not appear to prefer subadult females over adult females, perhaps because of a lower production of pheromones by the subadults that could make them harder for males to find [91,92]. *L. hesperus* males are also capable of detecting female hunger, which may prevent cannibalization. Information about female hunger is transmitted through pheromones in the web [93], and well-fed females tend to be more robust and larger than hungry females. In a laboratory study, *L. hesperus* males showed a clear preference for well-fed females [94], potentially decreasing cannibalism risk as female hunger is correlated with cannibalism rates [86]. Moreover, among females and juveniles, larger individuals tend to cannibalize smaller individuals [50], indicating different assessments used in different cannibalistic contexts. We were unable to find any studies discussing whether the females of any species use venom during these interactions, or if the use of venom differs between species where males invite cannibalism versus others. However, in *L. hasselti*, one of the species in which males self-sacrifice, males will die within two days of copulation, even if they survive the female’s initial attack [82], either to envenomation or injury. Comparing venom use during sexual cannibalism across these species could provide important insights into how spiders use social context in venom metering, a potential first step for the evolution of more complex social interactions and structures.

#### 4.1.2. Sibling Cannibalism

Another common type of cannibalism is sibling cannibalism. Even in many solitary species of spider, juveniles remain clustered on their mother’s web, dispersing in later instars. If food is scarce, they will begin either matriphagy (feeding off their mother) or cannibalizing their siblings for nutrients. Cannibalism rate varies between different clutches and maternal lineages, but follows a few common guidelines [40,41]. Cannibalization is typically size dependent, with larger spiders cannibalizing smaller, lower risk spiders. Accordingly, a low variance in spider size within a clutch will often decrease cannibalism rates [50,95]. Additionally, juveniles in the field often only aggregate with their siblings, but in the lab juveniles preferentially cannibalize unrelated juveniles, providing evidence for kin recognition (Section 2.1) [36]. As we do not currently know how venom develops over ontogeny, it is unclear if smaller spiders are “lower risk” due to low production of venom, capability of venom metering, or if their smaller body hinders them in an aggressive encounter.

#### 4.1.3. Avoiding Cannibalization

One way to mitigate the risk of cannibalism is communication. Invertebrates communicate using a mixture of several signal modes, including visual, seismic (vibratory), and chemical stimuli [96]. Web-spinning spiders use their webs to transmit vibrational signals [97,98]. These signals can be used to establish territory, indicate defensiveness or aggression (preventing the need to use costly venom) [99], and to find mates. In addition to transmitting vibrational signals, *Latrodectus* webs contain sex pheromones. Volatile pheromones act as attractants for male *Latrodectus*, indicating the position of webs as well as information about the female [100]. Once the male arrives on the web, he gains information from contact pheromones on the silk [101]. Contact sex pheromones also allow *Latrodectus* males to discriminate between webs of well-fed and hungry females, reducing their risk of cannibalism [93] (for more detail on sexual cannibalism, see Section 4.1). The transmission of both vibratory and pheromonal signals allow spiders to avoid risk, staying out of another individual’s territory, or away from a hungry female. The presence of these communication methods suggest potential ways for spiders to mitigate costly venom use if they can communicate risk prior to social interactions.

### 4.2. Social Learning

Spiders learn and modify their behavior through social interactions. An example of social learning can be seen in *Schizocosa ocreata* and *S. rovneri* (wolf spiders). Females of these species learn mating preference from exposure to males, with inexperienced females having no preference and experienced females preferring males with larger brushes [102]. Unfortunately, relatively few studies focus on learning in social spiders or other spiders like *Latrodectus*, although there are examples of early social environment influencing physiological, morphological, and behavioral traits later in life (developmental plasticity) [47]. For example, adult male *L. hasselti* resting metabolic rates differed when reared as juveniles with or without females and on different diets, resulting in differences in morphology and response to females [103]. The presence of developmental plasticity encourages further theory and research regarding this and other mechanisms potentially related to learning in spiders. Given the high-risk social system in *Latrodectus*, study of learning-related mechanisms could reveal new insights into how sociality is maintained in cannibalistic, venomous organisms and potentially shed light on the evolution of sociality in spiders.

One aspect of social learning in *Latrodectus* that remains unknown is the relationship between venom, cannibalization, and social learning. *Latrodectus* are known to cannibalize as juveniles, and it is often assumed that such cannibalization is venomous, though studies that address this specifically are scarce (Section 4.1). However, there are many interesting observations that have never been formally studied. For example, in the laboratory, *Latrodectus* spiderlings have a much lower mortality rate if they are separated from each other after two weeks, instead of immediately after hatch (L. Gatch, N. Singh, pers. obs.). This could be merely because they are fragile before this time (though this seems unlikely as they are observed to move and build webs properly after transfer), or there could be a potential benefit of being around their siblings, even with cannibalization occurring. This enhanced survival rate may be related to the advantage of being able to share prey. However, there is another intriguing possibility. While there is some evidence that snakes may learn how to properly meter venom for attacking prey by gaining experience hunting as juveniles [67], little is known about how spiders learn to meter their venom [66]. It is possible that the cannibalization of siblings is not simply a way of acquiring resources, but may allow juveniles to learn how to meter their venom on a more stationary target that does not require web use.

## 5. Conclusions

The relationship between venom and the evolution of complex social structures in invertebrates is an intriguing topic of study, yet has been primarily addressed in systems with complex, eusocial structures. Spiders provide an excellent system to compare venom use and evolution across taxa of varying social levels and social structures. Venom’s widespread use among invertebrates and vertebrates, and its effects on defense and social behavior, make its presence and evolution a potentially valuable tool for studying both social and venom evolution in a broader context.

## Data Availability

Not applicable.
